# SACE_5599, a putative regulatory protein, is involved in morphological differentiation and erythromycin production in *Saccharopolyspora erythraea*

**DOI:** 10.1186/1475-2859-12-126

**Published:** 2013-12-17

**Authors:** Benjamin Kirm, Vasilka Magdevska, Miha Tome, Marinka Horvat, Katarina Karničar, Marko Petek, Robert Vidmar, Špela Baebler, Polona Jamnik, Štefan Fujs, Jaka Horvat, Marko Fonovič, Boris Turk, Kristina Gruden, Hrvoje Petković, Gregor Kosec

**Affiliations:** 1 Acies Bio, d.o.o, Tehnološki park 21, SI-1000, Ljubljana, Slovenia; 2 Department of Biotechnology and Systems Biology, National Institute of Biology, Večna pot 111, SI-1000, Ljubljana, Slovenia; 3 Department of Biochemistry, Jožef Stefan Institute, Molecular and Structural Biology, Jamova cesta 39, SI-1000, Ljubljana, Slovenia; 4 Department of Food Science and Technology, University of Ljubljana, Biotechnical Faculty, Jamnikarjeva 101, SI-1000, Ljubljana, Slovenia; 5 Centre of Excellence for Integrated Approaches in Chemistry and Biology of Proteins, (CIPKeBiP), Jamova cesta 39, SI-1000, Ljubljana, Slovenia; 6 Center of Excellence of Nanosciences and Nanotechnology, Jamova cesta 39, SI-1000, Ljubljana, Slovenia; 7 Faculty of Chemistry and Chemical Technology University of Ljubljana, Aškerčeva cesta 5, SI-1000, Ljubljana, Slovenia; 8 Instituto de Biomedicina y Biotecnología de Cantabria (IBBTEC) Universidad de Cantabria, CSIC, SODERCAN, Facultad de Medicina, Avda. Cardenal Herrera Oria s/n, 39011, Santander, Spain

**Keywords:** *Saccharopolyspora erythraea*, Erythromycin, Polyketide, Regulator, SACE_5599, lmbU, Differentiation, Sporulation, Strain improvement, Metabolic engineering

## Abstract

**Background:**

Erythromycin is a medically important antibiotic, biosynthesized by the actinomycete *Saccharopolyspora erythraea*. Genes encoding erythromycin biosynthesis are organized in a gene cluster, spanning over 60 kbp of DNA. Most often, gene clusters encoding biosynthesis of secondary metabolites contain regulatory genes. In contrast, the erythromycin gene cluster does not contain regulatory genes and regulation of its biosynthesis has therefore remained poorly understood, which has for a long time limited genetic engineering approaches for erythromycin yield improvement.

**Results:**

We used a comparative proteomic approach to screen for potential regulatory proteins involved in erythromycin biosynthesis. We have identified a putative regulatory protein SACE_5599 which shows significantly higher levels of expression in an erythromycin high-producing strain, compared to the wild type *S. erythraea* strain. SACE_5599 is a member of an uncharacterized family of putative regulatory genes, located in several actinomycete biosynthetic gene clusters. Importantly, increased expression of SACE_5599 was observed in the complex fermentation medium and at controlled bioprocess conditions, simulating a high-yield industrial fermentation process in the bioreactor. Inactivation of SACE_5599 in the high-producing strain significantly reduced erythromycin yield, in addition to drastically decreasing sporulation intensity of the SACE_5599-inactivated strains when cultivated on ABSM4 agar medium. In contrast, constitutive overexpression of SACE_5599 in the wild type NRRL23338 strain resulted in an increase of erythromycin yield by 32%. Similar yield increase was also observed when we overexpressed the *bldD* gene, a previously identified regulator of erythromycin biosynthesis, thereby for the first time revealing its potential for improving erythromycin biosynthesis.

**Conclusions:**

SACE_5599 is the second putative regulatory gene to be identified in *S. erythraea* which has positive influence on erythromycin yield. Like *bldD*, SACE_5599 is involved in morphological development of *S. erythraea*, suggesting a very close relationship between secondary metabolite biosynthesis and morphological differentiation in this organism. While the mode of action of SACE_5599 remains to be elucidated, the manipulation of this gene clearly shows potential for improvement of erythromycin production in *S. erythraea* in industrial setting. We have also demonstrated the applicability of the comparative proteomics approach for identifying new regulatory elements involved in biosynthesis of secondary metabolites in industrial conditions.

## Introduction

Actinomycetes are an evolutionary diverse group of bacteria, predominantly soil-inhabiting organisms with GC-rich genomes and complex life cycles. Most actinomycetes are characterized by mycelial growth, multicellular behaviour, complex physiological and morphological differentiation and highly regulated biosynthesis of secondary metabolites with a broad spectrum of biological activities. Many of these natural products are of enormous industrial and clinical importance, e.g. as antiinfectives, anti-cancer agents and immunosuppressants. Soil-dwelling actinomycetes grow as vegetative mycelia of branching hyphae which explore the environment for available nutrients. When nutrients become scarce, for example after a few days of growth on an agar plate, aerial hyphae emerge from the colony into the air using the lysed vegetative mycelium as substrate [1,2]. Typically, antibiotic biosynthesis is initiated at this time [3]. Aerial hyphae then undergo several morphological stages, leading to subdivision of apical cells and finally to the release of spores [2]. These differentiation steps as well as biosynthesis of bioactive compounds (secondary metabolites) are coordinated by the complex regulatory pathways, which have been so far mostly studied in model actinomycetes, such as *Streptomyces coelicolor*. Important roles of some of the identified genes in differentiation, predominantly genes with regulatory function, have been elucidated based on the classical genetic studies. The obtained mutants, deficient in differentiation mainly fall into two phenotypic categories: mutants with “Bld” (bald) phenotype, which fail to make aerial mycelium and the “Whi” (white) mutants, which can form aerial mycelium but cannot produce spores and the spore pigment (reviewed in [2]).

*Saccharopolyspora erythraea* is an important filamentous actinomycete used in industrial fermentation processes for production of erythromycin, a medically important polyketide antibiotic. Semi-synthetic derivatives of erythromycin, such as clarithromycin and azithromycin, are widely used in clinical setting to treat infections caused by Gram-positive pathogens. In addition to its industrial importance, *S. erythraea* has been used as a model actinomycete system for studying the biosynthesis of secondary metabolites of polyketide origin, particularly the macrolide antibiotics, synthesized by modular type I polyketide synthase (PKS) genes [4,5]. Basic structure of the erythromycin biosynthetic gene cluster was found to span over 60 kbp of DNA and to comprise 20 genes transcribed in four main polycistronic units [6]. Three centrally located large PKS genes *eryAI*, *eryAII* and *eryAIII*, involved in the biosynthesis of macrolide core (6-deoxyerythronolide B), are flanked on both sides by the genes involved in post-PKS processing, biosynthesis and attachment of desosamine and mycarose sugar moieties to the erythronolide aglycone and erythromycin resistance [7,8]. Reflecting its industrial and scientific importance, the genome of *S. erythraea* wild type (WT) NRRL 23338 strain has been sequenced recently [8]. With the aim of achieving higher erythromycin productivity and fermentation processes scalable to industrial-scale bioreactors, the WT strain and the corresponding bioprocess have been improved for decades mainly by iterative methods of random mutagenesis and selection, resulting in industrial high-producing strains [9,10]. Due to very high industrial importance of erythromycin, the efforts towards improved *S. erythraea* strains are continuing by genetic/metabolic engineering as well as classical methods, assisted by genomic and transcriptomic approaches [9,11-13].

In most actinomycetes, timely and coordinated expression of large genetic clusters for polyketide biosynthesis, essential for efficient biosynthesis of the corresponding bioactive compounds, is achieved by several levels of regulatory control. First, pleiotropic (globally acting) regulatory genes, link signals from the environmental stimuli and life cycle progression to the biosynthetic machinery of the strain. Pleiotropic regulators are thought to activate transcription of pathway-specific regulatory genes, most often located inside biosynthetic gene clusters [14]. Pathway-specific regulators, such as the SARP (Streptomyces antibiotic regulatory protein) family regulators or the LAL (large ATP-binding regulators of the LuxR family) family regulators [15-17] then coordinate transcription of the biosynthetic genes most often located inside the corresponding clusters. In several actinomycetes engineering of regulatory mechanisms has shown a promising potential for increasing yields of natural products [18]. In some cases pathway-specific regulatory genes, naturally present in the biosynthetic gene clusters, have been overexpressed using strong constitutive promoters. In one example, overexpression of *tylS* and *tylR* regulatory genes in WT as well as in industrial overproducing strains of *Streptomyces fradiae* resulted in a significant increase in yields of tylosin, a macrolide antibiotic structurally related to erythromycin [19]. Similarly, yields of the immunosuppressants rapamycin and FK506 have been successfully increased by overexpression of the pathway-specific regulatory genes *rapG* (and *rapH*) in *Streptomyces rapamycinicus* and *fkbN* in *Streptomyces tsukubaensis*, respectively [20,21].

In striking contrast to the predominant regulatory cascade, present in most actinomycetes, the erythromycin gene cluster in *S. erythraea* does not contain pathway-specific regulatory genes, which has for some time precluded a better understanding of the regulatory mechanisms governing erythromycin biosynthesis. Importantly, microarray-based transcriptional studies revealed coordinated expression of most erythromycin biosynthetic genes and therefore the very likely existence of a common regulator for the *ery* cluster [12]. It was later demonstrated that this function can be carried out directly by BldD, a global regulatory protein with a key role in *S. erythraea* morphological differentiation. BldD was found to bind with a high affinity to all five promoter regions of the *ery* cluster, and the inactivation of the *bldD* gene reduced erythromycin production 7-fold in the WT strain of *S. erythraea*. In addition, *bldD* inactivation resulted in the Bld phenotype [22], analogously to the better characterized role of the *bldD* orthologue in *S. coelicolor*[23,24].

Based on a comparative proteomic approach and transcriptional analysis, carried out in the industrial cultivation conditions, we have identified a novel putative regulatory gene SACE_5599, which shows significantly higher expression levels in an industrial high producing strain of *S. erythraea*, ABE1441, compared to the WT strain. SACE_5599 is homologous to the putative regulatory genes located in several biosynthetic gene clusters of actinomycetes and its overexpression in *S. erythraea* NRRL23338 resulted in an enhanced erythromycin production (32% increased titre) at the shaker level, while its inactivation in the industrial strain led to significantly lower erythromycin yield, accompanied by a drastic reduction of sporulation on solid agar medium. Advancing our understanding of the regulation of erythromycin biosynthesis is of key importance as it will likely contribute towards increased efficiency of industrial fermentations of *S. erythraea* and potentially other industrial antibiotic-producing actinomycetes. In addition, this work demonstrates the potential of omics approaches for strain development in the industrial setting when analyses are carried out in industrially relevant conditions.

## Results

### Identification of differentially expressed protein SACE_5599

**Figure 1:**
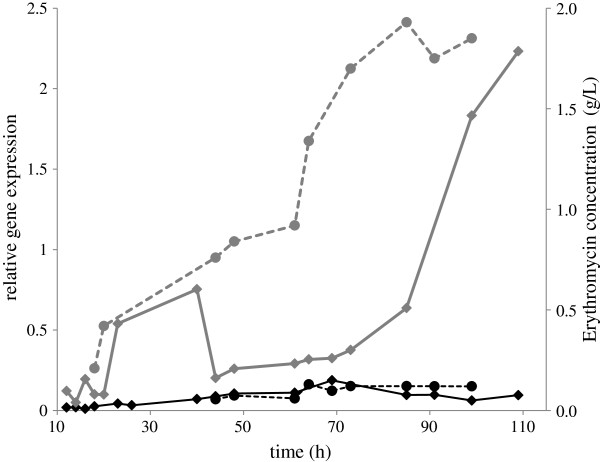
**Relative expression of SACE**_**5599 and erythromycin biosynthesis of the WT and industrial high**-**producing ABE1441 strains.** Data from one representative fermentation process are shown, normalized to expression of 16S rRNA. Grey lines represent relative expression of SACE_5599 () and erythromycin concentration () in the ABE1441 strain. Black lines represent relative expression of SACE_5599 () and erythromycin concentration () in the WT strain.

Regulatory mechanisms that control *S. erythraea* life cycle and erythromycin biosynthesis remain poorly characterized, which limits the genetic engineering efforts towards improved strains with increased yields of erythromycin. Based on the genome sequencing data more than 1000 genes with putative regulatory function are encoded in the *S. erythraea* genome [8]. However, their possible role in the life cycle or secondary metabolism is hard to predict based on the genome sequence alone. With the aim of identifying potential regulatory elements involved in regulation of erythromycin production during the fermentation process, we employed a comparative proteomic approach. We invested significant efforts to develop media from which high quality proteome and transcriptome data could be generated, at the same time ensuring relatively high yield of erythromycin, and maintaining key process conditions comparable with the industrial large scale fermentation process. Two *S. erythraea* strains, the WT NRRL23338 strain and an industrial erythromycin high-producing ABE1441 strain, developed from the WT strain through an intensive strain improvement program, were cultivated at 5 L bioreactor scale in medium and fermentation conditions simulating industrial fermentation process for production of erythromycin. In these conditions the NRRL23338 strain typically produces less than 100 mg/L erythromycin, whereas the ABE1441 strain produces approx. 2000 mg/L erythromycin (Figure 1). Representative samples of complete fermentation broths were collected at multiple time points during the erythromycin fermentation, cell-free protein extracts of *S. erythraea* were prepared and subjected to quantitative proteomic analysis. Relative quantification was performed using the spectral counting methodology, which compares numbers of detected MS/MS spectra for each of identified proteins [25].

**Figure 2:**
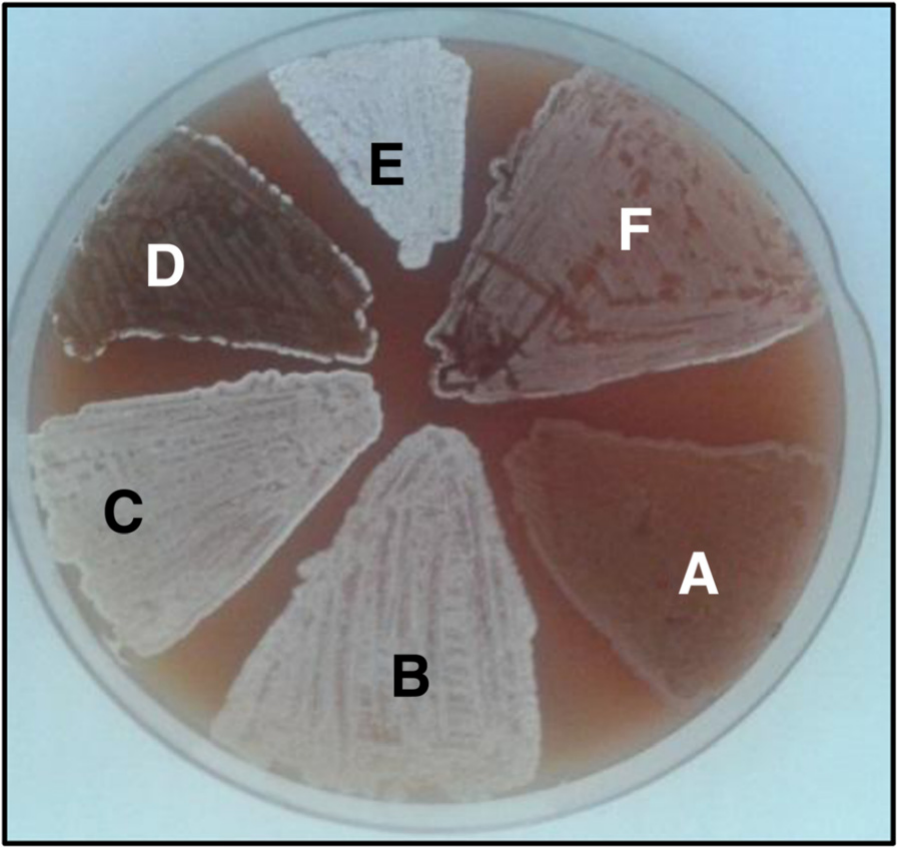
**Phenotypes of *S. erythraea* mutants with inactivated or *in trans* - overexpressed SACE_5599 on ABSM4 agar plates containing apramycin: A) NRRL23338/pSET152; B) ABE1441/pSET152; C) ABE1441/ΔSACE_5599 complemented with SACE_5599 (pABE110; pABE112); D) ABE1441/ΔSACE_5599 (pABE110); E) ABE1441/pSET152 + SACE_5599 (pABE104) F) NRRL23338/pSET152 + SACE_5599 (pABE104).** Construction of plasmid constructs is presented in Table 1. Equivalent result was observed when plates without apramycin were used (not shown).

In this way we aimed to identify regulatory proteins showing significant difference in expression levels at different stages (physiological conditions) of the fermentation process. It was our goal to obtain a small number of promising candidate regulatory proteins that could be further evaluated by gene inactivation and overexpression experiments with the final aim of increasing erythromycin yield through genetic engineering approaches. Interestingly, in addition to over 20-fold higher erythromycin yield achievable by the industrial strain, the WT and ABE1441 strains also differ significantly in intensity of sporulation on the ABSM4 and R5 agar media (Figure 2; Additional file 1), similarly to what has been previously observed for other erythromycin high-producing strains [9]. The high-producing strain ABE1441 showed abundant sporulation on ABSM4 agar plates (used in this study for inoculation of liquid media) whereas the NRRL23338 sporulates poorly and only after prolonged cultivation period.

**Figure 3:**
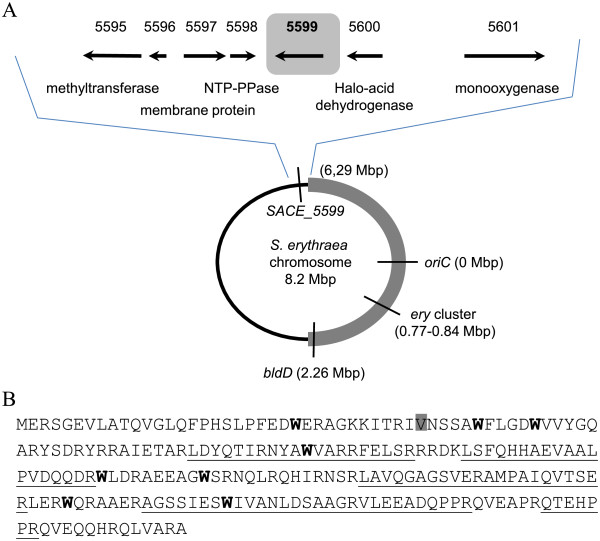
**Genomic context and amino acid sequence of SACE**_**5599. A)** Chromosomal locus of SACE_5599. Genes located close to SACE_5599 and their putative functions are indicated. SACE numbers are indicated above the arrows. SACE_5596 encodes a hypothetical protein with no similarity to other proteins in sequence databases. The position of SACE_5599 in *S. erythraea* genome is schematically represented. The »core« region is marked with bold grey line and the »non-core« region with thin black line. **B)** Sequence of the 219 amino acid ORF of SACE_5599 with peptides detected in proteomic analysis underlined, conserved tryptophan residues marked in bold and the position of alternative translation start (184 aa variant) is shaded in grey.

Among the proteins differentially expressed at the time of intense erythromycin biosynthesis (35 h for the WT and 27 h for the ABE1441 strain), SACE_5599 was identified as the putative regulatory protein with the most prominent difference in expression levels when comparing the industrial and WT strains. In fact, peptides derived from this protein were undetectable in protein extracts of the NRRL23338 WT strain, whereas several peptides of the SACE_5599 protein showed relatively high abundance in samples of the industrial ABE1441 strain (Figure 3B, Additional file 2).

### SACE_5599 is a member of a so far uncharacterized family of putative regulatory proteins

Based on these results it was our aim to investigate in more detail the putative regulatory gene SACE_5599 and its potential role in the regulation of erythromycin yield. Genomic analysis revealed that the SACE_5599 gene is located on the reverse strand at approx. position of 6.292 Mbp, inside the “non-core” region, believed to mainly contain genes related to conditionally adaptive functions [8]. Nevertheless, SACE_5599 lies very close to the limit of the predicted “core” region of *S. erythraea* chromosome [8] and is located 2.7 Mbp away from the *ery* cluster on 8.2 Mbp chromosome, surrounded by genes with no apparent role in erythromycin biosynthesis or secondary metabolism (Figure 3A). Similarly, the *bldD* (SACE_2077) regulator of transcription of erythromycin biosynthetic genes is also encoded relatively far away from the *ery* cluster and is located close to the other limit of “core” and “non-core” regions in the circular *S. erythraea* chromosome (Figure 3A). Interestingly, both the SACE_5599 and *bldD* lie approx. 2 Mbp from *oriC* in opposite directions. Importantly, the genome sequences of NRRL23338 and ABE1441 are identical inside the ORF and putative promoter region of SACE_5599 as well as of the adjacent genes (ŠF, HP, GK - unpublished results). SACE_5599 ORF was originally predicted at the time of *S. erythraea* genome annotation [8], however, an additional upstream start codon can also be envisioned, resulting in the length of the ORF of 184 or 219 amino acids, respectively (Figure 3B).

In addition to examining the genomic context, it was our aim to obtain more information about the possible function of SACE_5599 by identifying its potentially better characterized gene homologues in related bacterial species. A BlastP search [26] in the ‘nr’ database of NCBI identified a family of 29 thus far sequenced homologues with the E value < 10^-5^, nearly all of them located inside antibiotic gene clusters in the genomes of *Actinomycetales*. The length of predicted polypeptide chains of most homologues varies between 180 and 250 aa. The first member to be sequenced was the *lmbU* gene from the lincomycin biosynthetic gene cluster from *Streptomyces lincolnensis*. The *lmbU* gene contains a TTA codon in its nucleotide sequence and was therefore assigned a putative regulatory function [27]. The TTA leucine-encoding codon is extremely rare in actinomycetes and is often involved in synchronized regulation of expression of differentiation and secondary metabolism-related genes through regulated expression of the corresponding BldA-tRNA [2]. Sequence alignment of the homologues reveals a series of strikingly conserved tryptophan residues as well as numerous conserved arginine residues (arginine amounts to 14.2% of all amino acid residues of SACE_5599) in the N-terminal and central part of the protein (Figure 3B, Additional file 3). Theoretically predicted pI of the protein is 9.9, suggesting its possible function in nucleic acid binding. In addition, secondary structure prediction algorithms [28] predict a very high proportion of α-helical structure. Unfortunately, none of the homologues of SACE_5599 have been characterized so far at the biochemical or structural level, therefore little information is available on the roles and mechanism (s) of action of this family of proteins. Nevertheless, the notion of regulatory role of this family of proteins was further strengthened by gene-inactivation experiments of the *novE* homologue, located inside the biosynthetic gene cluster for novobiocin biosynthesis. When *novE*, also containing a TTA codon, was inactivated in *Streptomyces spheroides* NCIMB11891, the yield of novobiocin was reduced from 37 mg/L to 1.5 mg/L [29]. Interestingly, the TTA leucine codon is not present in the sequence of SACE_5599.

### qPCR analysis of SACE_5599 expression in NRLL 23338 and ABE1441 strains

In the next step we decided to compare in more detail the expression of SACE_5599 gene in the WT and erythromycin high-producing ABE1441 *S. erythraea* strains. We carried out a bioprocess for production of erythromycin at the scale of 5 L bioreactors, closely resembling industrial conditions, and samples for RNA analysis were harvested every 2 hours. Performing qPCR with SACE_5599-specific oligonucleotide primers we confirmed that transcription of SACE_5599 in the WT strain was very low throughout the bioprocess duration, peaking at around 70 h. In accordance with the results of the proteomic analysis, the expression of this gene was up to 20-fold higher in the high producing ABE1441 strain, particularly at the later stages of the cultivation process (Figure 1).

### Inactivation of SACE_5599 in the industrial overproducing strain of *S. erythraea* reduces erythromycin yield and has profound effect on morphological development

The observed differences in expression levels of SACE_5599 between the two strains suggest that this gene might be involved in the regulation of erythromycin biosynthesis of the ABE1441 overproducing strain and, possibly, also in the observed morphological differences between these two strains (Additional file 1). However, in order to establish whether there is a clear causative relationship between the drastically different expression levels of SACE_5599 and erythromycin yield we carried out the SACE_5599 gene inactivation in the industrial overproducing strain ABE1441. The pKC1132-based plasmid pABE110, containing 344 bp of the central region of SACE_5599 and not capable of replicating in *S. erythraea* was constructed and transferred into *S. erythraea* by conjugation from *E. coli*, thereby inactivating the putative regulatory gene by a single crossover gene disruption. The correct insertion through homologous recombination into the SACE_5599 locus and interruption of the ORF were confirmed by PCR and sequencing. 20 independent SACE_5599-inactivated ex-conjugants were obtained and all of them showed a morphologically modified phenotype, compared with the parent ABE1441 strain. On the ABSM4 solid medium, these mutant strains showed significantly reduced sporulation, which was also delayed for several days as compared to the original ABE1441 strain (Figure 2). Moreover, the mutant strains with inactivated SACE_5599 also produced substantially higher amounts of an unknown black pigment when grown on ABSM4 agar.

**Figure 4:**
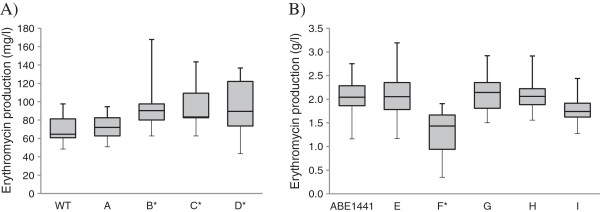
**Yield of erythromycin produced by different mutant strains of *S. erythraea.*** Bars encompass 95% of the sample population. Horizontal lines represent the median values and perpendicular lines indicate extreme values (min, max). Asterisks denote statistically significant differences between experimental group samples compared to control samples. The data were analyzed using SAS/STAT program as described in Methods. Panel **A)**: Erythromycin production by NRRL23338 transformants, determined by the microbiological assay; WT: control 1 NRRL23338; A: control 2 NRRL23338 + pSET152; B: NRRL23338 + SACE_5599-219 aa (pABE104); C: NRRL23338 + SACE_5599-219 aa-HA (pABE106); D: NRRL23338 + BldD (pABE21). Panel **B)**: Erythromycin production by industrial high-producing strain ABE1441 transformants, determined by the HPLC-UV method; ABE1441: control 1 ABE1441; E: control 2 ABE1441 + pSET152; F: ABE1441 ΔSACE_5599 (pABE110); G: ABE1441 ΔSACE_5599 + SACE_5599-219 aa-HA (pABE112) sporulating strains; H: ABE1441 + SACE_5599-219 aa (pABE104); I: ABE1441 + BldD (pABE21).

Further on, we tested the obtained mutants for erythromycin productivity in laboratory scale (shake flask level). Inactivation of the SACE_5599 gene resulted in a significant decrease (37%) in erythromycin yield in these mutant strains, compared to the original ABE1441 overproducing strain, which produced approx. 2.1 g/L erythromycin under the cultivation conditions used (Figure 4). Clearly, the decrease in erythromycin yield was not as drastic as to drop to the yield observed in the WT strain (around 60–70 mg/L) despite a complete lack of SACE_5599 expression in the SACE_5599-inactivated strains. Therefore, it can be concluded that the effect of differential expression of SACE_5599 on erythromycin yield in the high-producing ABE1441 strain seems to represent one of several factors, which contribute to increased erythromycin yield and likely form a complex interdependent regulatory/metabolic network. At this point it is not possible to speculate whether SACE_5599 is directly involved in transcriptional regulation of the *ery* genes or the effect of SACE_5599 on efficiency of erythromycin biosynthesis is indirect, possibly acting through regulation of so far unidentified metabolic/regulatory pathways, intimately associated with the differentiation process.

### Constitutive overexpression of SACE_5599 in the NRRL 23338 strain leads to erythromycin titre increase

Further on, it was our aim to evaluate whether overexpression of SACE_5599 in the WT *S. erythraea* strain can positively influence the regulatory network of the erythromycin biosynthetic processes and potentially lead to the phenotype more similar to the ABE1441 high producing strain, generated by strain improvement through random mutagenesis, thus leading to higher erythromycin yields and improved sporulation on ABSM4 agar medium. A set of pSET152-based expression vectors was constructed and used to integrate an additional copy of SACE_5599 in the *S. erythraea* chromosome. Relatively strong constitutive promoter PermE* was used in all cases to drive transcription *in trans*. As SACE_5599 has not been previously characterized or expressed *in trans*, eight different plasmid construct variants were prepared (Table 1). The variations were made in **a**) the 5’-UTR where variants with or without a ribosomal binding site (RBS) between the PermE* and the start codon were introduced, **b**) the SACE_5599 ORF to account for possible 184 aa or 219 aa ORF size, and **c**) the C-terminus of the protein where the HA-tag [30] was either absent or present, in order to be able to confirm the functional *in*-*trans* expression of SACE_5599 by western blotting. Each generated transformant was subjected to PCR analysis in order to confirm that the corresponding plasmid construct was integrated into *S. erythraea* genome by the action of ɸC31 integrase, encoded in the pSET152 backbone. Integration through homologous recombination into the native copy of SACE_5599 was not observed. In addition to the NRRL23338 WT strain, the same plasmid constructs were also introduced into the high producing ABE1441 strain in order to evaluate whether further increase in erythromycin yield can be achieved (see next section).

**Table 1:** Strains and plasmids used in this study

**Vector/strain name**	**Description**	**Promoter**	**Gene**	**Reference**
*S.erythraea*	WT- NRRL23338	/	/	[31,32]
*S.erythraea*	ABE1441	/	/	Acies Bio
pABE53	ɸC31, apramycin	PermE* without RBS	SACE_5599 (184 aa)	This work
pABE101	ɸC31, apramycin	PermE* with RBS	SACE_5599 (184 aa)	This work
pABE102	ɸC31, apramycin	PermE* without RBS	SACE_5599 (184 aa) HA-tag	This work
pABE103	ɸC31, apramycin	PermE* with RBS	SACE_5599 (184 aa) HA-tag	This work
pABE104	ɸC31, apramycin	PermE* without RBS	SACE_5599 (219 aa)	This work
pABE105	ɸC31, apramycin	PermE* with RBS	SACE_5599 (219 aa)	This work
pABE106	ɸC31, apramycin	PermE* without RBS	SACE_5599 (219 aa ) HA-tag	This work
pABE107	ɸC31, apramycin	PermE* with RBS	SACE_5599 (219 aa) HA-tag	This work
pABE110	Suicide, apramycin	/	344 bp long part of the SACE_5599 gene for disruption	This work
pABE21	ɸC31, apramycin	PermE* without RBS	*bldD* gene	This work
pABE112	ɸC31, thiostrepton	PermE* without RBS	SACE_5599 (219 aa ) HA-tag	This work

Site-specific integration with bacteriophage ɸC31 integrase is indicated for pSET152-derived plasmids; “/” refers to “none or not applicable”.

**Figure 5:**
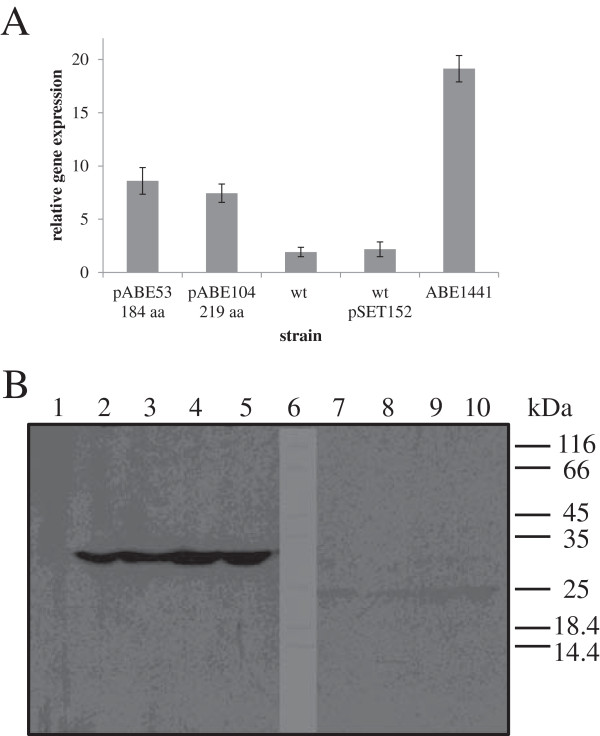
**Evaluation of *in***-***trans *expression of SACE**_**5599 in *S.erythraea. *A)** qPCR analysis of expression levels of SACE_5599 (normalized to expression of 16S rRNA) in WT and strains with additional copy of SACE_5599 (pABE53, pABE104), compared to the ABE1441 high-producing strain. **B)** Western blot analysis of *in trans* expression of 219 aa (lanes 2–5) and 184 aa (lanes 7–10) SACE_5599-HA and the control NRRL23338 strain with pSET152 (lane 1). Molecular mass markers were loaded in lane 6 and their positions are schematically presented on the right side of the blot. Bands of apparent molecular mass of 33 kDa are observed in 4 independent 219 aa transformants of the NRRL23338 strain, two containing the RBS sequence (pABE107) in the expression vector (lanes 2–3) and two without the RBS sequence (pABE106) (lanes 4–5). A very weak band of apparent molecular mass of 25 kDa was observed when shorter variant (184 aa) of SACE_5599 was expressed, either without RBS (plasmid pABE102 – lanes 7 and 8) or with RBS (plasmid pABE103 – lanes 9 and 10).

The obtained transformants of the NRRL23338 strain were then cultivated in shake flasks. In order to evaluate the suitability of the used vector/promoter system, cultures were sampled after 72 h of cultivation (estimated time of intense erythromycin biosynthesis in shake flasks) and SACE_5599 transcript levels were evaluated in strains transformed with different plasmids by qPCR. As shown in Figure 5A, qPCR analysis revealed significantly increased transcription levels in the transformants, constitutively overexpressing either 219 aa or 184 aa variant of SACE_5599, compared to the WT strain. Interestingly, transcription levels in transformants of the WT strain (with the second copy of SACE_5599 *in trans* under PermE* promoter) were still relatively low compared to the levels observed in the industrial high-producing ABE1441 strain, also determined in this qPCR analysis (Figure 5A). In order to confirm functional expression of the SACE_5599 protein, cell free protein extracts of the transformants with HA-tagged variants of SACE_5599 were subjected to western blot analysis. Importantly, we could detect strong specific bands of apparent molecular mass of (33 kDa) only in the samples in which longer variant of the gene was constitutively expressed, either without (pABE106) or with RBS (pABE107) in the 5’-UTR of the transcript (Figure 5B). In contrast, the bands corresponding to the shorter 184 aa variant (expressed from plasmids pABE102, pABE103) were observed at the molecular mass of 25 kDa but were very faint suggesting extremely low expression level. This suggests that the 219 aa variant of SACE_5599 protein is likely physiologically relevant. We hypothesize that the shorter 184 aa polypeptide variant might fold much less efficiently into a stable three-dimensional structure and is more rapidly degraded. Further on, similar intensity of the bands was observed in all 219 aa transformants, regardless of whether 5’-UTR with or without RBS was used (Figure 5B). The estimated molecular masses of both HA-tagged variants are slightly larger than their theoretically calculated values, possibly due to the very high pI of SACE_5599.

In the next experiment, erythromycin yield was determined in fermentation broths of the NRRL23338 strain transformants containing *in trans* copy of the longer 219 aa variant of SACE_5599 (plasmid pABE104) after 7 days of cultivation. Transformants containing the plasmid pABE106 with the HA-tagged version of SACE_5599 for which high overexpression was confirmed by western blot, were also included in the analysis. Erythromycin yield of the WT-based strains was determined by the microbiological assay based on *Bacillus subtilis* growth inhibition (see Methods), considering that at low erythromycin concentrations (below 200 mg/L) the erythromycin present in the broth cannot be reliably quantified by the used HPLC method. Indeed, erythromycin yield in SACE_5599 overexpressing strains was increased on average by 32% (Figure 4) compared to the WT level and addition of HA-tag to the C-terminus did not significantly influence the effect of SACE_5599 overexpression. It is noteworthy that the yield increase as well as observed expression levels of SACE_5599 are relatively low, although we used the constitutive PermE* promoter, which has previously been demonstrated as relatively strong promoter in *S. erythraea*[33]. Nevertheless, in this way we have clearly demonstrated a positive influence of SACE_5599 overexpression on erythromycin yield in the WT strain.

### Complementation of the SACE_5599-inactivated high-producing strain ABE1441

In parallel with *in*-*trans* expression in the NRRL23338 WT strain, we also introduced the 219 aa version of the gene SACE_5599 into the ABE1441 high producing strain, as well as into the SACE_5599-inactivated mutant of ABE1441. Interestingly, when SACE_5599 was over-expressed *in trans* in the high producing ABE1441 strain, using the plasmid pABE104, the yield of erythromycin was not significantly increased (Figure 4), suggesting that the underlying regulatory mechanisms have already been optimized (improved) in this industrial strain through random mutagenesis and selection procedures. Further on, one of the 20 SACE_5599-inactivated strains of ABE1441 high producing strain (see above) was complemented with the longer 219 aa variant of the gene. In this case, a thiostrepton resistance cassette was added to the pABE106 vector, which was used for selection, because the apramycin resistance marker is already present in the plasmid used for inactivation of the native SACE_5599 copy (Table 1). Normal sporulation phenotype, observed in the parent ABE1441 strain, was restored in about 80% of the ex-conjugants, which had SACE_5599 disrupted strain and then complemented *in trans*. Interestingly, erythromycin productivity was restored to the levels of the ABE1441 high-producing strain, which coincided with the recovery of original sporulation capacity (Figure 4). In contrast, in strains which failed to regain sporulation capacity, erythromycin yield also remained at the level of SACE_5599-inactivated mutants. It is important to stress that decreased yields of the strains which did not sporulate on ABSM4 agar medium, were not caused by poor growth/biomass formation, as evaluated by the pH and packed cell volume (PCV) measurements of vegetative media before inoculating the production media (not shown). Therefore, the effect of SACE_5599 on erythromycin biosynthesis seems to be closely related to its role in morphological differentiation, similarly to what was observed in the case of the *bldD* gene [22].

### Moderate increase in erythromycin yield is achieved by overexpression of BldD in *S. erythraea* NRRL23338

It was one of important objectives of our work to evaluate the biotechnological potential of regulatory genes, known to influence erythromycin yield, in an effort to obtain further improved *S. erythraea* strains. Therefore, in parallel with overexpression of the newly identified SACE_5599, we also overexpressed in the NRRL 23338 strain the *bldD* gene, for which the positive regulatory role in erythromycin biosynthesis was demonstrated earlier [22]. For expression of *bldD* we used the same vector-promoter system (plasmid pABE21) as described above for expression of SACE_5599 and fermentation was carried at shake flask level as described above (Table 1). Indeed, *bldD*-overexpressing strains produced on average approx. 30% more erythromycin compared to the wild type strain (Figure 4), resulting in similar yield improvement as observed with SACE_5599 overexpression. Interestingly, when *bldD* was over-expressed *in trans* in the high producing ABE1441 strain, the yield of erythromycin was not further increased, but in contrast, was slightly decreased compared to the original ABE1441 strain (Figure 4).

## Discussion

Improving the understanding of regulatory elements involved in erythromycin biosynthesis in *S. erythraea* remains a challenging task because no regulatory genes are present inside the erythromycin gene cluster. Analogously as demonstrated for *bldD*[22], other potential regulators influencing erythromycin biosynthesis, might be located in other regions of the chromosome of *S. erythraea* and affect multiple physiological processes simultaneously, e.g. morphological differentiation or other metabolic pathways, in addition to transcriptional control of erythromycin biosynthetic genes. The difficulty of identifying key regulatory genes, crucial for improvement of erythromycin yield, is reflected by the fact that among about 7000 predicted ORFs in *S. erythraea* genome 15.5% are putative regulatory genes [8]. Even a recent comparative genomic analysis of the natural and industrial erythromycin over-producing strain identified 40 genomic variations affecting genes related to the regulation of transcription and translation processes [9].

In this work we have identified a putative regulatory gene/protein SACE_5599, based on a comparative proteomic analysis of erythromycin high-producing ABE1441 and WT *S. erythraea* strains, cultivated in industrially based medium. After *bldD*, SACE_5599 is only the second putative regulatory gene identified to date to affect erythromycin yield. This finding shows a promising potential of the non-gel based proteomic techniques to detect and identify differentially expressed regulatory proteins, which are generally of extremely low abundance. The results of gene inactivation and overexpression experiments carried out in this work showed that this gene is indeed involved in the increased yield of erythromycin in the industrial high-producing ABE1441 strain. In addition to decreasing erythromycin yield, inactivation of SACE_5599 also resulted in the morphological phenotype with drastically reduced sporulation of the resulting mutant strains. While levels of expression of SACE_5599 are clearly related to erythromycin biosynthesis, an important question remains about the mechanism of action of this putative regulatory protein as well as of its homologues in other actinomycetes. Considering the high predicted pI value of SACE_5599 and reports of putative regulatory roles of its homologues in other actinomycetes it can be speculated that SACE_5599 acts as transcriptional regulator by binding to specific DNA/RNA sequences [29].

Interestingly, a profound effect on morphological development of *S. erythraea* was observed when *bldD*, the first identified regulatory protein involved in the regulation of erythromycin biosynthesis , was inactivated [22]. Our results thus support the idea that the regulatory network involved in morphological differentiation interconnects very closely with erythromycin biosynthesis. Moreover, the propensity of aerial mycelium formation seems to correlate well with erythromycin production among spontaneously rifampicin-resistant mutants of the NRRL23338 strain [34], suggesting that from the regulation aspect, morphological differentiation and erythromycin biosynthesis are very closely related phenomena. In another recent study, the *bld* phenotype of *bldD*-inactivated strains was shown to be overcome by inactivation of the SACE_7040 gene, a regulator of the TetR family. Unfortunately, it was not reported whether re-established morphological phenotype also led to the return of original erythromycin yields in that double mutant strain [35]. Another recent study revealed the role of the SACE_0012 gene in morphological differentiation of *S. erythraea*, however, in this case, no significant change in erythromycin productivity was observed in the SACE_0012-inactivated strains in spite of the observed early aerial hyphae formation of this strain [36].

In an attempt to analyse how our results correlate with the previous work of other groups in *S. erythraea* and to gain a more comprehensive insight into the roles of SACE_5599 and *bldD*, we took a closer look at the data of recent high-throughput studies, carried out with *S. erythraea*. In particular, we analysed how the transcription of these two regulatory genes correlated with erythromycin biosynthesis at different phases of cultivation. In most studies in the past, cultivation of *S. erythraea* was carried out in soluble non-industrial media. In contrast, we made particular effort to develop media and bioprocess parameters closely resembling the industrial conditions. Interestingly, the SACE_5599 gene was one of the few genes that were not included in the microarray design of most previous transcriptomic studies [9,12,34,37], therefore no information is available about its expression profiles in these studies. In contrast, transcription levels of *bldD* were studied in most of the published studies. In an initial attempt to characterize transcriptional changes in *S. erythraea* WT strain during different growth phases, the expression *bldD* (SACE_2077) was found to decrease during transition from initial growth phase (phase A) to growth slowdown phase (phase B), similarly to expression of erythromycin biosynthetic genes [37]. However, when the same transcriptome data was later reinterpreted the *bldD* gene was not categorized into the same co-transcriptional module as the erythromycin biosynthetic cluster [38]. Interestingly, when different rifampicin-resistant mutants of *S. erythraea* were later profiled by DNA-microarrays, *bldD* transcription levels were lower in strains with higher transcription of erythromycin biosynthetic genes and increased erythromycin yield [34]. Moreover, in this study *bldD* levels were higher in strains with decreased erythromycin yield, thereby not suggesting a close link between the BldD expression and erythromycin yield. In contrast, a spontaneous streptomycin-resistant mutant containing a mutation in the *rpsL* gene (encoding the S12 ribosomal protein) showed a markedly increased erythromycin yield which correlated with an increased BldD expression at the late stages of the bioprocess [39]. In a further comparative study transcriptional profiles of the NRRL23338 and an erythromycin overproducing Px strain were evaluated [9]. In this study the erythromycin-overproducing strain showed higher levels of *bldD* transcription compared to the WT in the initial phases of the fermentation process. However, after 35 h *bldD* transcript levels were comparable in both strains, whereas most prominent differences in erythromycin productivity between the two strains were observed between the 48 h and 72 h time point. An RNASeq analysis of *S. erythraea* transcriptome was also carried out recently, which permitted us to analyse expression of SACE_5599 as well as *bldD*. Indeed, transcription of some of the genes from the erythromycin cluster was found to correlate well with SACE_5599, while no correlation was apparent with *bldD*[40]. During the preparation of this manuscript, another comparative genomic and transcriptomic analysis of the WT and high-producing *S. erythraea* E3 strain was reported [41]. Importantly, industrial growth media were used for *S. erythraea* cultivation in this study and SACE_5599 was included in the microarray design. Similarly to our observations SACE_5599 showed significantly higher expression levels in the high producing strain, compared to the WT in all time points of the fermentation process. In contrast BldD expression was significantly decreased in the high producing strain.

In summary, different studies report different degree of correlation between expression levels of either *bldD* or SACE_5599 and erythromycin biosynthesis. There are at least three possible reasons for these discrepancies. Firstly, it is reasonable to expect that the preparation of total RNA or construction of microarrays can vary significantly, thereby influencing the outcome on transcriptional analysis. Secondly, different media and cultivation conditions were applied, which clearly have effect on secondary metabolism and differentiation. Finally, the discrepancies between different studies might be related to the fact that due to the lack of pathway-specific regulation, erythromycin biosynthesis is regulated directly through globally acting regulatory elements which at the same time also control morphological differentiation of *S. erythraea*. Therefore, differential expression of multiple regulatory genes might play a key role in increased erythromycin yield in each individual high-producing strain, considering that every strain was developed through an independent media and process development in combination with classical mutagenesis and selection programs. Further experiments are necessary in order to elucidate regulatory mechanisms by which SACE_5599 as well as *bldD* can affect erythromycin yield. Interestingly, after the regulatory network of BldD orthologue has been studied in significant detail in *Streptomyces coelicolor*, the actinomycete model organism [23,24,42,43], a more complex relationship between BldD expression and erythromycin biosynthesis in *S. erythraea* has been recently suggested [24].

Regardless of the poorly understood underlying regulatory and metabolic pathways, the *S. erythraea* production strain and bioprocess for production of erythromycin have been improved for over 50 years. A significant increase in erythromycin yields has been achieved, compared to the WT strain. However, considering the annual world production and commercial importance of erythromycin and its semi-synthetic derivatives, current yields remain relatively low in comparison to other antibiotic production technologies, e.g. tetracyclines or penicillin [44]. Therefore, there is a clear commercial incentive to further improve erythromycin production technology. In addition to the classical methods of random mutagenesis and selection, which have been already exhausted to great extent, metabolic and biosynthetic engineering are now expected to have a key role in future improvement strategies. For example, engineering the methylmalonyl-CoA mutase (mcm) enzymatic activity has been shown to have good potential for increasing the flux through the feeder pathway of methylmalonyl-CoA, the main precursor of the erythromycin polyketide chain. Inactivation of mcm lead to yield increase in carbohydrate-based medium and overexpression of the mcm operon enabled significant yield increase in an oil based medium [11,45].

To our knowledge, improvement of erythromycin titres by manipulation of expression of regulatory genes has so far not been demonstrated. In this work we show that using a comparative proteomic approach combined with an extra effort to cultivate *S. erythraea* in conditions simulating the industrial setting, regulatory genes relevant for increasing erythromycin yield in genetic engineering approaches can be identified. Interestingly, SACE_5599 could also have been envisioned as a possible candidate gene based on a very recent comparative transcriptomics study, also carried out industrial media [41]. Our results thus open new possibilities for increasing erythromycin yields by overexpressing globally acting (pleiotropic) regulatory genes involved in key processes of development and differentiation. Specifically, approaches aimed at achieving higher levels or optimum temporal control of *in trans* expression of SACE_5599 could result in further improved yields. Further on, other candidate regulatory genes in *S. erythraea* genome, identified by recent omics analyses, carried out in industrial conditions should be evaluated for their potential of further improvement of the yield. Particularly, regulators providing further yield increase in industrial high-producing strains would be desirable for biotechnological applications. In this way, manipulation of expression of regulatory genes will become a complementary strategy to manipulation of metabolic genes which has already led to significant improvements of erythromycin yields.

## Conclusions

In conclusion, we have identified a new putative regulatory gene SACE_5599 in *S. erythraea* that influences sporulation during the life cycle of this actinomycete and importantly affects erythromycin yield of the WT and high-producing *S. erythraea* strains. While the detailed regulatory mechanisms of this protein remain to be elucidated, we have observed that erythromycin biosynthesis and final yield in *S. erythraea* are strongly interrelated with regulatory networks involved in morphological differentiation. We have also shown that overexpression of SACE_5599 and *bldD* gene (SACE 2077) can improve the yield of erythromycin in the *S. erythraea* WT strain, suggesting that modulation of expression of globally acting regulators may lead to improved producers of polyketide natural products. Importantly we have shown, that omics approaches are valuable tools to identify industrially relevant regulatory genes, however, it is of key importance that samples for omics analysis are collected in conditions which simulate real industrial setting.

## Materials and methods

### Cultivation and transformation of *S. erythraea* strains

The *S. erythraea* NRRL23338 type strain and ABE1441 industrial overproducing strain, obtained by iterative methods of classical mutagenesis and selection, were propagated on ABSM4 agar plates (1% corn starch, 1.1% corn steep liquor, 0.3% (NH4)_2_SO_4_, 0.3% NaCl, 0.3% CaCO_3_, 2% agar) or R5 agar plates [46] for 14-days at 30°C. Laboratory scale fermentation in liquid culture was carried out for estimation of erythromycin productivity. Seed cultures were prepared in the ABVM1 medium (3% corn steep liquor, 3% sucrose, 0.4% (NH_4_)_2_SO_4_, 0.6% CaCO_3_) at 30°C and 220 rpm for 48 h at 6 mL or 40 mL of working volume in Falcon tubes or Erlenmeyer flasks. Erythromycin production phase was carried out in the ABPM8 medium (3.6% soybean flour, 3.6% corn starch, 0.24% (NH_4_)_2_SO_4_, 0.72% CaCO_3_, 0.5% soybean oil) inoculated with 10% (v/v) of the above seed culture. At time of inoculation 2% glucose and 0.67% n-propanol were added. Cultivations were performed at 5 mL or 25 mL working volume at 30°C and 220 rpm for 7 days. After 24 h of cultivation 1% glucose and 0.34% n-propanol were added. Apramycin (50 μg/ml) and thiostrepton (25 μg/ml solid and 5 μg/ml liquid media) were added to the solid and liquid media as required.

Seed cultures for the cultivation in bioreactors were prepared as described above, using 400 ml of the ABVM1 seed medium in 2 L Erlenmeyer flasks. Fermentation experiments were carried out in 5 L Bioreactors (Sartorius) with 3.5 L of ABPM8 production medium, operated at 30°C, 1 vvm airflow and 350–900 rpm agitation. Bioreactors were inoculated with 10 vol.% seed culture. Dissolved oxygen concentration and pH were monitored using autoclavable electrodes (Hamilton). Dissolved oxygen was maintained above 20% with increasing agitation and aeration rate during the bioprocess. Foaming was controlled by automatic addition of antifoam. Samples for RT-PCR and proteomic analysis were taken during the bioprocess and stored at -80°C until analysis as described below.

Transformation of *S. erythraea* strains was achieved by conjugation of pSET152 [46] and pKC1132 [47] based plasmid constructs from the *E. coli* strain ET12567 carrying the *E. coli*-*Streptomyces* conjugation facilitating plasmid pUZ8002, as described previously [48]. Standard methods for isolation and manipulation of DNA were used [32,46].

### Bioinformatic methods

BlastP searches [26] were performed against the nr protein database of NCBI (http://www.ncbi.nlm.nih.gov) using amino acid sequence of SACE_5599 as query. Hits with E value < 10^-5^ were considered to be homologous and were aligned using ClustalW2 program with default parameters (http://www.ebi.ac.uk).

### Comparative proteomic analysis

Whole fermentation broth samples were centrifuged and washed three times with 50 mM Tris–HCl, pH = 7.2. Cells were resuspended in lysis buffer containing 7 M urea, 2 M tiourea, 4% CHAPS, 40 mM Tris, 65 mM DTT and complete protease inhibitor cocktail (Roche) and subsequently sonicated. Homogenate was centrifuged to obtain cell extract. Proteins in the cell extract were separated on a 12% precast SDS-PAGE gel (Lonza) and stained using Coomassie Brilliant Blue dye. Whole protein lanes were excised, sliced into bands and subjected to the standard trypsin digestion procedure as previously described [49]. Peptides were extracted from the gel, and injected into nanoLC HPLC unit (Proxeon) coupled with Orbitrap LTQ Velos mass spectrometer (Thermo Scientific). Peptides were separated on a Picofrit C18 analytical column (New Objective) with a 90 minute linear gradient of 5-50% acetonitrile/0.1% formic acid. MS/MS spectra were generated using CID fragmentation of precursor ions generated in the MS scans.

Protein identification and relative quantification by spectral counting was carried out using MaxQuant database search software [50] and species specific NCBI database. For the database search Methionine oxidation was set as variable and carbamidomethylation of cysteines was set as fixed modification.

### Quantitative real time PCR (qPCR) analysis

Expression of SACE_5599 gene was monitored in the time course of the fermentation process in WT, ABE1441 and in overexpression strains. For SACE_5599 primers (F: CTGGATCGTGGCCAACCT, R: GGGCGCCTCGACCTG) and TaqMan MGB probe (FAM- CTGGTCGGCTTCCTCC -MGB) were designed by Life Technologies (Assay by Design service). 16S rRNA primers, targeting SACE_8105, (F: ACTGAGACACGGCCCAGACT, R: AGGCTTKCGCCCATTGTG) and TaqManMGB probe (FAM-CTGCTGCCTCCCGTAG-MGB), designed in-house using Primer Express (Life Technologies), were used for normalization.

RNA was isolated from 0.5 ml of fixated (ethanol:phenol 96:4) culture. After removal of the fixative, the pellet was treated with lysozyme (Sigma, 15 mg/ml) and proteinase K (6000U, Qiagen) in TE buffer for 10 minutes, followed by homogenization with a bead beater (FastPrep, Qbiogene) using Lysing Matrix B (Qbiogene) beads and RLT Buffer (550 μl, Qiagen). Lysates were centrifuged and supernatant was used for subsequent RNA isolation using RNeasy Mini Kit (Qiagen, Hilden, Germany) with following modifications of the manufacturer’s protocol: samples were washed with RW1 and RPE buffer twice and three-times, respectively. RNA was eluted with pre-heated (65°C) RNAse-free water following 5 minute incubation. To remove remaining genomic DNA, total RNA samples were treated in solution with Qiagen’s RNase-free DNase digestion kit (Qiagen, 0.5 U/μg RNA). Efficiency of DNase digestion was tested using RT^-^ controls. RNA quantity and quality was checked using Nanodrop, gel electrophoresis and Bioanalyzer (Agilent) Reverse transcription and qPCR analysis of SACE_5599 and 16S rRNA was performed using LightCycler 480 as described previously [51]. The standard curve method was used for relative gene expression quantification, and the transcript accumulation of SACE_5599 was normalized to 16S rRNA.

### Overexpression of SACE_5599 and *bldD* genes

219 aa and 184 aa variants of SACE_5599 gene (Figure 3B) were amplified using *S. erythraea* genomic DNA as template. The shorter gene variant was amplified using the following primers: forward primer AAAATATCATATGAATTCGTCGGCCTGGTTCCTCGGTGAC introducing NdeI restriction site into the start codon and reverse primers AAAAATCTAGAATTATGCCCGGGCGACCAGTTGCCGGTGCT and AAAAATCTAGAATTAGGCGTAGTCCGGGACGTCGTACGGGTATGCCCGGGCGACCAGTTGCCGGTGCT for HA-tagging of the protein, in both cases introducing XbaI restriction site after the stop codon. The 219 aa gene variant was amplified using the following forward primer: AAAAACATATGGAACGCTCCGGTGAGGTGCTGGCG and the same reverse primers used as for the 184 aa variant of the gene. The PCR amplified genes were cloned into pSet152 vector into which the constitutive PermE* promoter [52] was previously cloned, thus creating series of pABE vectors (Table 1). The identity of the PCR products was confirmed by sequencing. As the 5’-UTR of the native *ermE* gene lacks obvious RBS, a variant of the promoter sequence containing RBS [21] was also assembled to achieve constitutive expression of SACE_5599.

The *bldD* gene was amplified using *S. erythraea* genomic DNA as template using the following primers: forward primer AAAAAAACATATGGGCGACTACGCCAAGGCGCTGGG introducing NdeI restriction site into the start codon and reverse primer AAATCTAGACTCACTCCTCCCGGGCCGGGCGC introducing XbaI restriction site after the stop codon. As above, the PCR amplified gene was cloned into pSet152 vector with PermE* promoter and confirmed by sequencing. The obtained plasmid constructs were introduced into *S. erythraea* NRRL23338 strain using standard conjugation procedure [46] and the insertion of plasmid constructs into pseudo att site was confirmed by PCR. The obtained independent colonies were cultivated according to the above procedures and erythromycin yields were estimated by the microbiological assay.

### Inactivation of SACE_5599 in the ABE1441 strain

Gene disruption of SACE_5599 was carried out by transformation of the ABE1441 strain with pKC1132 [47] based plasmid construct containing an 344 bp central region of the SACE_5599 gene (pABE110). The central region of SACE_5599 was PCR amplified using the following primers: d5599F AAAGAATTCGTCGGCCTGGTTCCTCGGTGAC containing EcoRI restriction site at 5’-end and d5599R AAAAAAGCTTTGGTCACCTGGATGGCGGGCATC containing HindIII restriction site at 5’-end. The PCR fragment was gel purified, digested with EcoRI and HindIII enzymes and cloned into pKC1132 vector opened with the same enzymes to generate pKC1132-d5599 plasmid construct (pABE110). The correct nucleotide sequence was confirmed by sequencing. The pKC1132-d5599 was transformed into the ABE1441 strain using standard conjugation procedure described previously [46] and SACE_5599 gene was inactivated through a single-crossover recombination event. Inactivation of SACE_5599 in 20 independent apramycin-resistant ex-conjugants was confirmed by PCR and sequencing. The obtained independent colonies were cultivated according to the above procedures and erythromycin yields were estimated by HPLC.

Complementation of SACE_5599 in the mutant strain *S. erythraea* OP (ΔSACE_5599) was carried out by insertion of vectors containing the 219 aa variant of the gene under the control of PermE* promoter. The pABE112 vectors was constructed by inserting the thiostrepton resistance marker [46] into the MscI restriction site of the corresponding pSET152-derived vector (pABE106) containing the 219 aa variant of the SACE_5599 gene.

### Detection and quantification of erythromycin in fermentation broths

#### HPLC

After cultivation was completed, the pH of the broth was first adjusted to 9.5-10 and the broth was mixed with the equal volume of acetonitrile (1:1) for 40 min. After that 1 g NaCl was added per 5 g of broth, left to dissolve and the acetonitrile phase was then separated by centrifugation and applied onto the Nucleodur HTec C18, 3 μm (150 × 4.6 mm, Macherey-Nagel, Dueren, Germany) reversed-phase stationary phase HPLC column installed on the Thermo Finnigan Surveyor + HPLC system. Isocratic elution was applied with mobile phase prepared from 20% 50 mM K_2_HPO_4_ with pH adjusted to 9 with diluted phosphoric acid and 80% acetonitrile. After injection of 10 ul of the sample solution, the HPLC system was operated at a flow rate of 1 ml / min, with total the run time of 30 minutes. The column temperature was set at 60°C and the detection wavelength at 206 nm. Reference substance erythromycin A was obtained from the European Pharmacopoeia and was 98.4% pure. Standard solution was prepared by dissolving 5 mg of erythromycin in 5 mL of solvent. All chemicals used were HPLC-grade.

### Growth inhibition assay

The titres of erythromycin produced by *S. erythraera* NRRL23338 strain and its genetically modified variants were determined using a conventional Pharmacopoeia bioassay method (European Pharmacopoeia 5.0). As standard commercially available eythromycin (Calbiochem) was used and *Bacillus subtilis* NRRL B-765 strain was grown as test organism. Samples were extracted in acetonitrile and 60 μL of properly diluted extracts were transferred in cylinder halls on ABA agar test medium (Biolife) which contained overnight *Bacillus subtilis* culture. The plates were incubated for 14 – 16 h at 37°C. Thereafter, sizes of inhibition zones were measured and erythromycin concentrations calculated according the standard curve. The method has a linear response in the range from 1 mg/L to 20 mg/L and all samples were diluted accordingly.

### Statistical analysis

Yields of erythromycin were calculated with SAS/STAT software using means and the univariate procedure to test the normality of distribution. Using the GLM model, data were calculated as least mean square and are presented as an average change observed from all experiments when comparing least mean square values to the wild-type control least mean square value of each experiment.

### SDS PAGE and western blotting

Cell-free protein extracts of *S. erythraea* were prepared by washing the cells in 100 mM K-phosphate buffer, pH = 7.4 twice, resuspending in the same buffer containing complete protease inhibitor cocktail (Roche) and subsequent sonification. A total of 50 μg of proteins were boiled in Laemmli’s sample buffer. Proteins were separated on a 15% SDS-PAGE gel and transferred to a nitrocellulose membrane. Immunodetection was performed with monoclonal rat anti-HA primary antibodies (Roche) and horseradish peroxidase conjugated anti-rat secondary antibodies (Calbiochem). The antigens were visualized with chemoluminiscence detection system (GE Healthcare).

## Abbreviations

Aa: Amino acid; Bp: Base pair; GC-rich: Containing high proportion of guanosine and cytosine bases; Mcm: Methylmalonyl-CoA mutase; ORF: Open reading frame; PCV: Packed cell volume; RBS: Ribosomal binding site; UTR: Untranslated region; WT: Wild type.

## Competing interests

JH, ŠF, HP and GK are shareholders in Acies Bio, Ltd.

## Authors’ contributions

BK and VM carried out cultivation of *S. erythraea* in shaker and fermenter level with sampling and analysed the morphology of transformant strains. VM, MT and MH carried out molecular cloning, conjugation of *S. erythraea*, genetic analysis of ex-conjugants and western blot analyses. KK and PJ participated in preparation of cell extracts for comparative proteomic analysis. JH set up and carried out analytical methods for erythromycin determination. ŠB analysed qPCR data, MP interpreted the data in the context of other high-throughput studies, MP, ŠB and KG participated in study design and helped to draft the manuscript. RV, MF and BT performed mass spectrometry analysis and proteomic data processing and evaluation. ŠF was involved in study design, data interpretation and statistical analysis. HP and GK were involved in the conceptualization and design of the study, data interpretation and manuscript preparation. All authors read and approved the final manuscript.

## Supplementary Material

Additional file 1: Figure S1 Differences in sporulation intensity of the *S. erythraea* strains on ABSM4 (A) and R5 (B) agar medium (Kieser et al., 2000 [46]). The ABE1441 strain is plated on the upper half and the NRRL23338 WT strain is plated on the lower half of both plates. Click here for file

Additional file 2: Table S1 Proteomic identification of SACE_5599 in erythromycin overproducing ABE1441 strain. Table A shows the list of identified peptides with their corresponding peptide mass, Posterior Error Probability (PEP) and Maxquant peptide score. Table B shows peptide spectral counts obtained from the analysis of the WT (NRRL23338) and industrial strain (ABE1441). Experiment was done in two biological replicates. Click here for file

Additional file 3: Figure S2 Protein sequence alignment of SACE_5599 family of proteins (ClustalW2). Conserved tryptophan residues are marked in red and conserved arginine residues are marked in blue. Central region of the protein, showing particularly high sequence similarity among all homologues, is marked in grey. Homologues from selected *Actinomycetales* species are presented: Slin – LmbU from *S. lincolnensis*; W007 – LmbU from *Streptomyces* sp. W007; Sery – SACE_5599 from *S. erythraea*; Shim – HmtD from *Streptomyces himastatinicus* ATCC53653 ; Stsu – *Streptomyces tsukubaensis* NRRL18488; Faln – *Frankia alni* ACN14a; K744 – Orf10 from *Kutzneria* sp. 744; Scae – NovE from *Streptomyces spheroides*; Sgri – HrmB from *Streptomyces griseoflavus*; Scla – *Streptomyces clavuligerus* ATCC27064. Click here for file

## References

[B1] Chater KF Regulation of sporulation in Streptomyces coelicolor A3(2): a checkpoint multiplex? Curr Opin Microbiol 2001 12 6 667 673 10.1016/S1369-5274(01)00267-3 11731318

[B2] McCormick JR Flardh K Signals and regulators that govern *Streptomyces* development FEMS Microbiol Rev 2012 12 1 206 231 10.1111/j.1574-6976.2011.00317.x 22092088 PMC3285474

[B3] van Wezel GP McDowall KJ The regulation of the secondary metabolism of Streptomyces: new links and experimental advances Nat Prod Rep 2011 12 7 1311 1333 10.1039/c1np00003a 21611665

[B4] Donadio S Staver MJ McAlpine JB Swanson SJ Katz L Modular organization of genes required for complex polyketide biosynthesis Science 1991 12 5006 675 679 10.1126/science.2024119 2024119

[B5] Cortes J Haydock SF Roberts GA Bevitt DJ Leadlay PF An unusually large multifunctional polypeptide in the erythromycin-producing polyketide synthase of *Saccharopolyspora erythraea* Nature 1990 12 6297 176 178 10.1038/348176a0 2234082

[B6] Reeves AR English RS Lampel JS Post DA Vanden Boom TJ Transcriptional organization of the erythromycin biosynthetic gene cluster of *Saccharopolyspora erythraea* J Bacteriol 1999 12 22 7098 7106 10559177 10.1128/jb.181.22.7098-7106.1999 PMC94186

[B7] Weber JM Leung JO Maine GT Potenz RH Paulus TJ DeWitt JP Organization of a cluster of erythromycin genes in *Saccharopolyspora erythraea* J Bacteriol 1990 12 5 2372 2383 2185216 10.1128/jb.172.5.2372-2383.1990 PMC208872

[B8] Oliynyk M Samborskyy M Lester JB Mironenko T Scott N Dickens S Haydock SF Leadlay PF Complete genome sequence of the erythromycin-producing bacterium *Saccharopolyspora erythraea* NRRL23338 Nat Biotechnol 2007 12 4 447 453 10.1038/nbt1297 17369815

[B9] Peano C Tala A Corti G Pasanisi D Durante M Mita G Bicciato S De Bellis G Alifano P Comparative genomics and transcriptional profiles of *Saccharopolyspora erythraea* NRRL 2338 and a classically improved erythromycin over-producing strain Microb Cell Fact 2012 12 32 10.1186/1475-2859-11-32 22401291 PMC3359211

[B10] Zhang Q Wu J Qian J Chu J Zhuang Y Zhang S Liu W Knocking out of tailoring genes eryK and eryG in an industrial erythromycin-producing strain of *Saccharopolyspora erythraea* leading to overproduction of erythromycin B, C and D at different conversion ratios Lett Appl Microbiol 2011 12 2 129 137 10.1111/j.1472-765X.2010.02973.x 21175699

[B11] Reeves AR Brikun IA Cernota WH Leach BI Gonzalez MC Weber JM Engineering of the methylmalonyl-CoA metabolite node of *Saccharopolyspora erythraea* for increased erythromycin production Metab Eng 2007 12 3 293 303 10.1016/j.ymben.2007.02.001 17482861 PMC2722834

[B12] Lum AM Huang J Hutchinson CR Kao CM Reverse engineering of industrial pharmaceutical-producing actinomycete strains using DNA microarrays Metab Eng 2004 12 3 186 196 10.1016/j.ymben.2003.12.001 15256208

[B13] Chen Y Deng W Wu J Qian J Chu J Zhuang Y Zhang S Liu W Genetic modulation of the overexpression of tailoring genes eryK and eryG leading to the improvement of erythromycin A purity and production in *Saccharopolyspora erythraea* fermentation Appl Environ Microbiol 2008 12 6 1820 1828 10.1128/AEM.02770-07 18223111 PMC2268306

[B14] Bibb MJ Regulation of secondary metabolism in streptomycetes Curr Opin Microbiol 2005 12 2 208 215 10.1016/j.mib.2005.02.016 15802254

[B15] Wietzorrek A Bibb M A novel family of proteins that regulates antibiotic production in streptomycetes appears to contain an OmpR-like DNA-binding fold Mol Microbiol 1997 12 6 1181 1184 10.1046/j.1365-2958.1997.5421903.x 9350875

[B16] De Schrijver A De Mot R A subfamily of MalT-related ATP-dependent regulators in the LuxR family Microbiology 1999 12 Pt 6 1287 1288 10411254 10.1099/13500872-145-6-1287

[B17] Rascher A Hu Z Viswanathan N Schirmer A Reid R Nierman WC Lewis M Hutchinson CR Cloning and characterization of a gene cluster for geldanamycin production in *Streptomyces hygroscopicus* NRRL 3602 FEMS Microbiol Lett 2003 12 2 223 230 10.1016/S0378-1097(02)01148-5 12586396

[B18] Martin J-F Liras P Engineering of regulatory cascades and networks controlling antibiotic biosynthesis in *Streptomyces* Curr Opin Microbiol 2010 12 3 263 273 10.1016/j.mib.2010.02.008 20303823

[B19] Stratigopoulos G Bate N Cundliffe E Positive control of tylosin biosynthesis: pivotal role of TylR Mol Microbiol 2004 12 5 1326 1334 10.1111/j.1365-2958.2004.04347.x 15554972

[B20] Kuscer E Coates N Challis I Gregory M Wilkinson B Sheridan R PetkoviÄ‡ H Roles of rapH and rapG in positive regulation of rapamycin biosynthesis in *Streptomyces hygroscopicus* J Bacteriol 2007 12 13 4756 4763 10.1128/JB.00129-07 17468238 PMC1913445

[B21] Goranovic D Blazic M Magdevska V Horvat J Kuscer E Polak T Santos-Aberturas J Martinez-Castro M Barreiro C Mrak P FK506 biosynthesis is regulated by two positive regulatory elements in *Streptomyces tsukubaensis* BMC Microbiol 2012 12 238 10.1186/1471-2180-12-238 23083511 PMC3551636

[B22] Chng C Lum AM Vroom JA Kao CM A key developmental regulator controls the synthesis of the antibiotic erythromycin in *Saccharopolyspora erythraea* Proc Natl Acad Sci U S A 2008 12 32 11346 11351 10.1073/pnas.0803622105 18685110 PMC2516264

[B23] Elliot MA Bibb MJ Buttner MJ Leskiw BK BldD is a direct regulator of key developmental genes in *Streptomyces coelicolor* A3(2) Mol Microbiol 2001 12 1 257 269 10.1046/j.1365-2958.2001.02387.x 11298292

[B24] den Hengst CD Tran NT Bibb MJ Chandra G Leskiw BK Buttner MJ Genes essential for morphological development and antibiotic production in *Streptomyces* coelicolor are targets of BldD during vegetative growth Mol Microbiol 2010 12 2 361 379 10.1111/j.1365-2958.2010.07338.x 20979333

[B25] Li Z Adams RM Chourey K Hurst GB Hettich RL Pan C Systematic comparison of label-free, metabolic labeling, and isobaric chemical labeling for quantitative proteomics on LTQ Orbitrap Velos J Proteome Res 2012 12 3 1582 1590 10.1021/pr200748h 22188275

[B26] Altschul SF Wootton JC Gertz EM Agarwala R Morgulis A Schäffer AA Yu Y-K Protein database searches using compositionally adjusted substitution matrices FEBS J 2005 12 20 5101 5109 10.1111/j.1742-4658.2005.04945.x 16218944 PMC1343503

[B27] Peschke U Schmidt H Zhang HZ Piepersberg W Molecular characterization of the lincomycin-production gene cluster of Streptomyces lincolnensis 78–11 Mol Microbiol 1995 12 6 1137 1156 10.1111/j.1365-2958.1995.tb02338.x 8577249

[B28] Cole C Barber JD Barton GJ The Jpred 3 secondary structure prediction server Nucleic Acids Res 2008 12 W197 W200 Web Server issue 10.1093/nar/gkn238 18463136 PMC2447793

[B29] Eustaquio AS Luft T Wang Z-X Gust B Chater KF Li S-M Heide L Novobiocin biosynthesis: inactivation of the putative regulatory gene novE and heterologous expression of genes involved in aminocoumarin ring formation Arch Microbiol 2003 12 1 25 32 10.1007/s00203-003-0555-2 12736771

[B30] Pati UK Novel vectors for expression of cDNA encoding epitope-tagged proteins in mammalian cells Gene 1992 12 2 285 288 10.1016/0378-1119(92)90589-H 1376293

[B31] Bunch RL McGuire JM Office USPaT Erythromycin, its salts, and method of preparation 1953 USA

[B32] Sambrook J Russell DW Molecular Cloning: A Laboratory Manual 2001 3 Cold Spring Harbor Laboratory: Cold Spring Harbor, NY

[B33] Brunker P Minas W Kallio PT Bailey JE Genetic engineering of an industrial strain of *Saccharopolyspora erythraea* for stable expression of the *Vitreoscilla* haemoglobin gene (vhb) Microbiology 1998 12 Pt 9 2441 2448 9782491 10.1099/00221287-144-9-2441

[B34] Carata E Peano C Tredici SM Ferrari F Tala A Corti G Bicciato S De Bellis G Alifano P Phenotypes and gene expression profiles of *Saccharopolyspora erythraea* rifampicin-resistant (rif) mutants affected in erythromycin production Microb Cell Fact 2009 12 18 10.1186/1475-2859-8-18 19331655 PMC2667423

[B35] Han S Song P Ren T Huang X Cao C Zhang B Identification of SACE_7040, a member of TetR family related to the morphological differentiation of *Saccharopolyspora erythraea* Curr Microbiol 2011 12 2 121 125 10.1007/s00284-011-9943-z 21626147

[B36] Yin X Xu X Wu H Yuan L Huang X Zhang B SACE_0012, a TetR-Family Transcriptional Regulator, Affects the Morphogenesis of *Saccharopolyspora erythraea* Curr Microbiol 2013 10.1007/s00284-013-0410-x PMC3825060 23793130

[B37] Peano C Bicciato S Corti G Ferrari F Rizzi E Bonnal RJ Bordoni R Albertini A Bernardi LR Donadio S Complete gene expression profiling of *Saccharopolyspora erythraea* using GeneChip DNA microarrays Microb Cell Fact 2007 12 37 10.1186/1475-2859-6-37 18039355 PMC2206050

[B38] Chang X Liu S Yu YT Li YX Li YY Identifying modules of coexpressed transcript units and their organization of *Saccharopolyspora erythraea* from time series gene expression profiles PLoS One 2010 12 8 e12126 10.1371/journal.pone.0012126 20711345 PMC2920828

[B39] Tanaka Y Komatsu M Okamoto S Tokuyama S Kaji A Ikeda H Ochi K Antibiotic overproduction by rpsL and rsmG mutants of various actinomycetes Appl Environ Microbiol 2009 12 14 4919 4922 10.1128/AEM.00681-09 19447953 PMC2708438

[B40] Marcellin E Mercer TR Licona-Cassani C Palfreyman RW Dinger ME Steen JA Mattick JS Nielsen LK *Saccharopolyspora erythraea*’s genome is organised in high-order transcriptional regions mediated by targeted degradation at the metabolic switch BMC Genomics 2013 12 15 10.1186/1471-2164-14-15 23324121 PMC3610266

[B41] Li Y-Y Chang X Yu W-B Li H Ye Z-Q Yu H Liu B-H Zhang Y Zhang S-L Ye B-C Systems perspectives on erythromycin biosynthesis by comparative genomic and transcriptomic analyses of *S. erythraea* E3 and NRRL23338 strains BMC Genomics 2013 12 1 523 10.1186/1471-2164-14-523 23902230 PMC3733707

[B42] Elliot MA Locke TR Galibois CM Leskiw BK BldD from *Streptomyces coelicolor* is a non-essential global regulator that binds its own promoter as a dimer FEMS Microbiol Lett 2003 12 1 35 40 10.1016/S0378-1097(03)00474-9 12900018

[B43] Kelemen GH Viollier PH Tenor J Marri L Buttner MJ Thompson CJ A connection between stress and development in the multicellular prokaryote *Streptomyces coelicolor* A3(2) Mol Microbiol 2001 12 4 804 814 10.1046/j.1365-2958.2001.02417.x 11401688

[B44] Demain AL Adrio JL Strain improvement for production of pharmaceuticals and other microbial metabolites by fermentation Prog Drug Res 2008 12 251 253 289 10.1007/978-3-7643-8117-2_7 18084918

[B45] Reeves AR Brikun IA Cernota WH Leach BI Gonzalez MC Weber JM Effects of methylmalonyl-CoA mutase gene knockouts on erythromycin production in carbohydrate-based and oil-based fermentations of *Saccharopolyspora erythraea* J Ind Microbiol Biotechnol 2006 12 7 600 609 10.1007/s10295-006-0094-3 16491356

[B46] Kieser T Bibb MJ Buttner MJ Chater KF Hopwood DA Practical Streptomyces genetics 2000 Norwich, United Kingdom: The John Innes Foundation

[B47] Bierman M Logan R O’Brien K Seno ET Rao RN Schoner BE Plasmid cloning vectors for the conjugal transfer of DNA from *Escherichia coli* to *Streptomyces* spp Gene 1992 12 1 43 49 10.1016/0378-1119(92)90627-2 1628843

[B48] Paget MS Chamberlin L Atrih A Foster SJ Buttner MJ Evidence that the extracytoplasmic function sigma factor sigmaE is required for normal cell wall structure in Streptomyces coelicolor A3(2) J Bacteriol 1999 12 1 204 211 9864331 10.1128/jb.181.1.204-211.1999 PMC103550

[B49] Zhang J Fonovic M Suyama K Bogyo M Scott MP Rab35 controls actin bundling by recruiting fascin as an effector protein Science 2009 12 5945 1250 1254 10.1126/science.1174921 19729655

[B50] Cox J Mann M MaxQuant enables high peptide identification rates, individualized p.p.b.-range mass accuracies and proteome-wide protein quantification Nat Biotechnol 2008 12 12 1367 1372 10.1038/nbt.1511 19029910

[B51] Petek M Baebler S Kuzman D Rotter A Podlesek Z Gruden K Ravnikar M Urleb U Revealing fosfomycin primary effect on *Staphylococcus aureus* transcriptome: modulation of cell envelope biosynthesis and phosphoenolpyruvate induced starvation BMC Microbiol 2010 12 159 10.1186/1471-2180-10-159 20515462 PMC2887449

[B52] Bibb MJ Janssen GR Ward JM Cloning and analysis of the promoter region of the erythromycin resistance gene (ermE) of *Streptomyces erythraeus* Gene 1985 12 1–3 215 226 2998943 10.1016/0378-1119(85)90220-3

