# Supplementary material for: SACE_5599, a putative regulatory protein, is involved in morphological differentiation and erythromycin production in Saccharopolyspora erythraea
Source: Microb Cell Fact. 2013 Dec 17;12:126. doi: 10.1186/1475-2859-12-126 (PMC3878487; doi:10.1186/1475-2859-12-126)
Supplement: Additional file 1: Figure S1 — Differences in sporulation intensity of the S. erythraea strains on ABSM4 (A) and R5 (B) agar medium (Kieser et al., 2000 [46]). The ABE1441 strain is plated on the upper half and the NRRL23338 WT strain is plated on the lower half of both plates. [file 1475-2859-12-126-S1.pptx]

## Slide 1
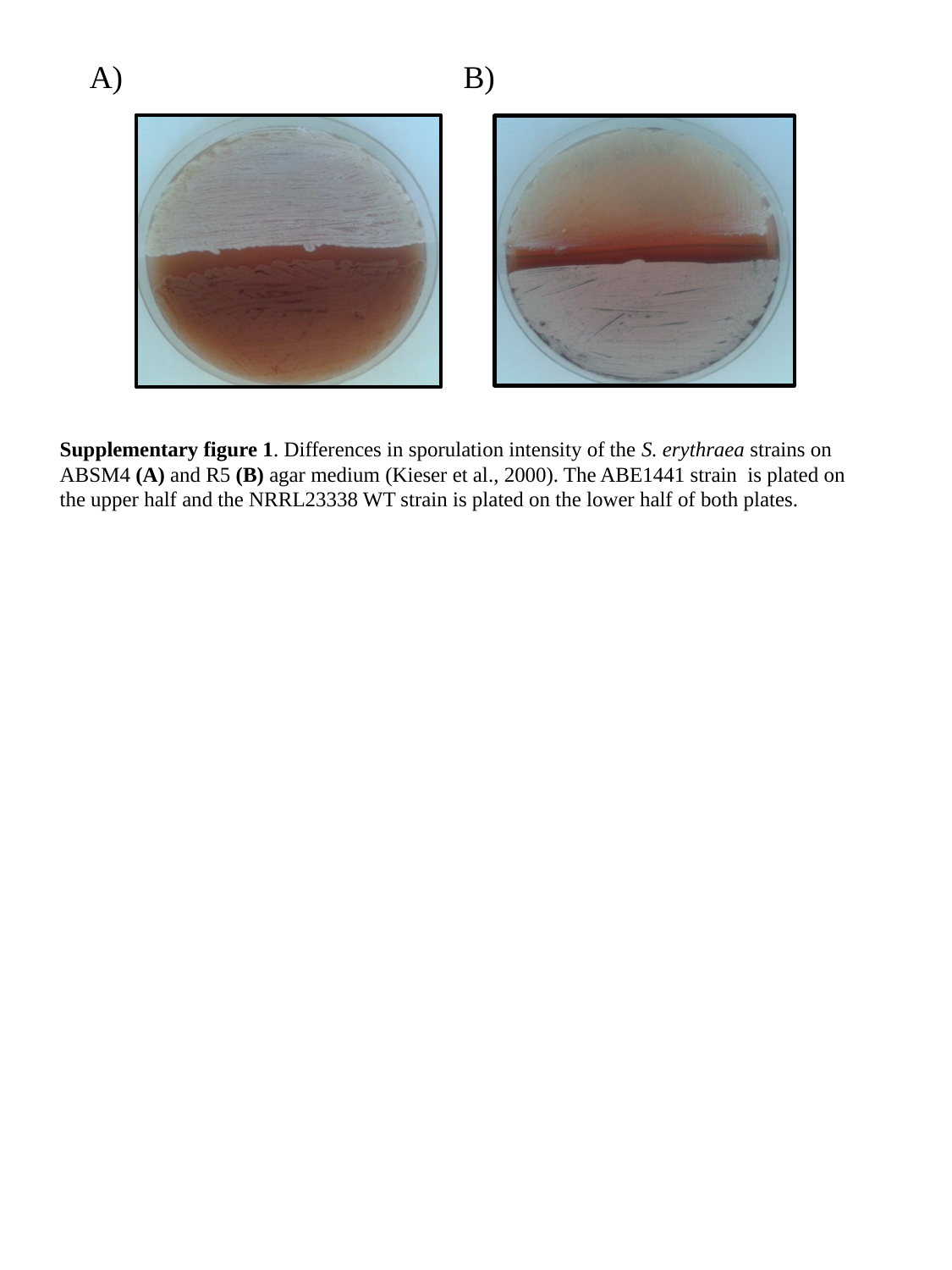

A)
B)
Supplementary figure 1. Differences in sporulation intensity of the S. erythraea strains on ABSM4 (A) and R5 (B) agar medium (Kieser et al., 2000). The ABE1441 strain is plated on the upper half and the NRRL23338 WT strain is plated on the lower half of both plates.
