# Supplementary material for: SACE_5599, a putative regulatory protein, is involved in morphological differentiation and erythromycin production in Saccharopolyspora erythraea
Source: Microb Cell Fact. 2013 Dec 17;12:126. doi: 10.1186/1475-2859-12-126 (PMC3878487; doi:10.1186/1475-2859-12-126)
Supplement: Additional file 2: Table S1 — Proteomic identification of SACE_5599 in erythromycin overproducing ABE1441 strain. Table A shows the list of identified peptides with their corresponding peptide mass, Posterior Error Probability (PEP) and Maxquant peptide score. Table B shows peptide spectral counts obtained from the analysis of the WT (NRRL23338) and industrial strain (ABE1441). Experiment was done in two biological replicates. [file 1475-2859-12-126-S2.docx]

Supplementary table 1

A

| Peptide sequence | Peptide mass (Da) | PEP | Peptide score |
| --- | --- | --- | --- |
| AGSSIESWIVANLDSAAGR | 1902.944 | 3.45E-36 | 290.62 |
| AMPAIQVTSER | 1201.613 | 0.00236 | 114.97 |
| LAVQGAGSVER | 1085.583 | 0.000197 | 136.63 |
| LDYQTIR | 907.4763 | 0.1243 | 128.75 |
| LSFQHHAEVAALPVDQQDR | 2160.071 | 9.42E-11 | 157.22 |
| NYAWVAR | 878.4399 | 0.11711 | 118.01 |
| QTEHPPR | 863.425 | 0.1405 | 81.32 |
| RFELSR | 806.4399 | 0.17487 | 107.22 |
| VLEEADQPPR | 1152.578 | 0.001196 | 133.13 |

B

|  | MS/MS count strain NRRL23338 | MS/MS count strain ABE1441 |
| --- | --- | --- |
| Biological replicate 1 | 0 | 26 |
| Biological replicate 2 | 0 | 47 |

**SupplementaryTable 1. Proteomic identification of SACE_5599 in erythromycin overproducing ABE1441 strain**

Table A shows the list of identified peptides with their corresponding peptide mass, Posterior Error Probability (PEP) and Maxquant peptide score. Table B shows peptide spectral counts obtained from the analysis of the WT (NRRL23338) and industrial strain (ABE1441). Experiment was done in two biological replicates.
