# Supplementary material for: SACE_5599, a putative regulatory protein, is involved in morphological differentiation and erythromycin production in Saccharopolyspora erythraea
Source: Microb Cell Fact. 2013 Dec 17;12:126. doi: 10.1186/1475-2859-12-126 (PMC3878487; doi:10.1186/1475-2859-12-126)
Supplement: Additional file 3: Figure S2 — Protein sequence alignment of SACE_5599 family of proteins (ClustalW2). Conserved tryptophan residues are marked in red and conserved arginine residues are marked in blue. Central region of the protein, showing particularly high sequence similarity among all homologues, is marked in grey. Homologues from selected Actinomycetales species are presented: Slin – LmbU from S. lincolnensis; W007 – LmbU from Streptomyces sp. W007; Sery – SACE_5599 from S. erythraea; Shim – HmtD from Streptomyces himastatinicus ATCC53653 ; Stsu – Streptomyces tsukubaensis NRRL18488; Faln – Frankia alni ACN14a; K744 – Orf10 from Kutzneria sp. 744; Scae – NovE from Streptomyces spheroides; Sgri – HrmB from Streptomyces griseoflavus; Scla – Streptomyces clavuligerus ATCC27064. [file 1475-2859-12-126-S3.docx]

**Slin**  -----------------MVRSNLSVADRCGTSAVNGRVKTGEDGVLVTRVGLRIPAVLNF 43

**W007**  --------------------------------------------MLATRVGLRISAGLDY 16

**Sery**  --------------------- ----------------MERSGEVLATQVGLQFPHSLPF 22

**Shim**  ------MMNQSSDLDRPKVVLFGKRPD-AERQRRTGPADTRG-QVLTTNVGLRIPVGLTF 52

**Stsu**  --------------------------------------------MLTTKVGLQMPAQLAY 16

**Faln**  -----MMMNQIEGRENSDVLTSRAEPEGVPRARRPGGQGPRLPQVLTTKVGLHIPPGLAF 55

**K744**  ------MGRGMTVSNSVSYSASAMVRDRHAGARPPQQRHPSDERILTTKNGLHIPEGLSF 54

**Scae**  MVASGRTASKGRGNGATPVRPTAGDATPVDSGQPSDTTYGGLE-VSAERTRLRIPRDLSL 59

**Sgri**  -----------MHNAMT-----AGDGARVLSGGGGPEALG-----EVERTSLRLPRDMTI 39

**Scla**  MERYGQYRSARRTGAAPHGPPGGPNGVERGGARSLQNRLGITDGVYAGQVELRIPAGLPF 60

**Slin**  DTWERAGRHIARVADSSAWCLGDWIIYGQTRYSDRYRRAVEAAGLDYQTIRNYAWVARRF 103

**W007**  EVWERAGQRIARVADSSAWCLGDWIIYGESRYTDRYRRAVKAAGLDYQTIRNYAWVARSF 76

**Sery**  EDWERAGKKITRIVNSSAWFLGDWVVYGQARYSDRYRRAIETARLDYQTIRNYAWVARRF 82

**Shim**  DDWERAGRQLSSIVNSSSWWLGDWLVYGKHHYTDRYQRGIRTAGLQYQTLRNYAWVSRRF 112

**Stsu**  DEWERSGRQLAGVLDSSSWWLGDWLVYGKDHYTDRYQRGIRAVGLSYQTLRNYAWVARRF 76

**Faln**  EDWERAGRQLSGLIDSSSWWLGDWLIYGKDQYVDRYERGIRAAGLQYQTLRNYSWVCRRF 115

**K744**  SEWKVAGRRLSGILDSSCWWLGDWLVFGKEQYSDRYQRGVEAVNLSYQTLRNYAWVARRF 114

**Scae**  EAWCRLGGRILAVCDSSVWWIGDWLVFGQNQYGDRYRRAMKETKLDYQTLRNYAWVARKF 119

**Sgri**  AAWQRLGERIAGLSDSSAWWIGDWLVFGQERFPDRYKRAMAETTLDYQTLRNYAWVARRF 99

**Scla**  DSWCRLGSQIKRVSESSVWWLADWLVFGEEEYPDRYQVVIKRTSLSYQTLRNYAWVARKF 120

**Slin**  DLSRRREALSFQHHAEVAALPEEQQDHWLEQAERHEWSRNELRRNVRGARGQKKSDTRRD 163

**W007**  DQSRRRSSLSFQHHAEVAALAPEQQDYWLDQSKRFGWSRNELRKNVRGAQ-RGKPAPAAV 135

**Sery**  ELSRRRDKLSFQHHAEVAALPVDQQDRWLDRAEEAGWSRNQLRQHIRNSR-LAVQGAGSV 141

**Shim**  ELHRRRPALTFQHHAELASLSIEEQDQWLDRAEQMGWTTKQLRHALRLAR-EDDINHGRA 171

**Stsu**  DLTRRRSALSFQHHAELASMPVEEQDFWLDRAEQRQWTTKQLRGALRAAR-RGEEPPKAP 135

**Faln**  DMPRRRSALSFQHHAELASLPVDEQDAWLDRAEHLKWTTKQLRNAVRAGR-QRVAGPTST 174

**K744**  PLERRRAQLSFQHHSEIASLPDDEQEQWLDEAEARSWTTKQLRSAVRMAQ-HNGLSEGDA 173

**Scae**  EPSRRRDSLTFQHHMEVAALSEAEQDHWLDFAVRLNWSRNELRKQIRASLSGEEDD--LR 177

**Sgri**  VPDRRCAGLTFQHHMEVAALTEEEQDHWLDFASRLSWSRNELRKQVRASRESADEK--DE 157

**Scla**  PVARRRDALSLQHHAETAALPADEQDRWLERAERERWSARRLRRELRG-HHAVDGG--AA 177

**Slin**  ADVLSRITPEAERVERWRTAAERSGASLEEWICARLDFAASLVLQTQAE-----DAR--- 215

**W007**  PEAVPQIKASPERVQRWRLAAERSGNTLEGWISARLDAAAAVMLG-LAQ-----DAP--- 186

**Sery**  ERAMPAIQVTSERLERWQRAAERAGSSIESWIVANLDSAAGRVLEEADQ-----PPRQVE 196

**Shim**  TEPTRQLAVPGNRLQWWHKAAELSGTDLEQWVLATLDRAAERALEEVVE-----EPQEIS 226

**Stsu**  AGPSRRLEVPGNRLQWWHRAAEQSGIAFDQWVMTTLDSAAAHALEEPGGPQEPREPREIP 195

**Faln**  AIAPRALVLPDHRLQRWHTAAIQVGSELSAWVTTTLDAAASQVLKDRKA-----EPELPR 229

**K744**  S--MKQLAVPGSRVKCWRQAAERAGVAFEQWVLSTLDHAAELELDSESG-----PPDTQL 226

**Scae**  CEVQLNLQLDELRLERWREAARGSNLTLTDWILSVVDGAV-------------------- 217

**Sgri**  SEVRLSVRIAQDRLDRWEAAARKSSTTLTEWMGAVLDRAV-------------------- 197

**Scla**  ARVQVTLNLDSERRGRWTQAAEAAHVPLMEWIVRTVDDAAAP------------------ 219

**Slin**  -------------REAGEAVGGA---------------------------- 225

**W007**  -------------ANR----------------------------------- 189

**Sery**  APRQTEHPPRQVEQQHRQLVARA---------------------------- 219

**Shim**  G-------------------------------------------------- 227

**Stsu**  GRGETGGPAGPDGGVPDTAVAGADAPDAGGYGVSARTAGLAGQAPPAVVGA 246

**Faln**  A-------------------------------------------------- 230

**K744**  GNGGEEDGYADRTG------------------------------------- 240

**Scae**  ---------------------------------------------------

**Sgri**  ---------------------------------------------------

**Scla**  ---------------------------------------------------

**Supplementary figure 2**. Protein sequence alignment of SACE_5599 family of proteins (ClustalW2). Conserved tryptophan residues are marked in red and conserved arginine residues are marked in blue. Central region of the protein, showing particularly high sequence similarity among all homologues, is marked in grey. Homologues from selected *Actinomycetales* species are presented: **Slin** – LmbU from *S. lincolnensis*; **W007** – LmbU from *Streptomyces* sp. W007; **Sery** – SACE_5599 from *S. erythraea*; **Shim** – HmtD from *Streptomyces himastatinicus* ATCC53653 ; **Stsu** – *Streptomyces tsukubaensis* NRRL18488; **Faln** – *Frankia alni* ACN14a; **K744** – Orf10 from *Kutzneria* sp. 744; **Scae** – NovE from *Streptomyces spheroides*; **Sgri** – HrmB from *Streptomyces griseoflavus*; **Scla** – *Streptomyces clavuligerus* ATCC27064
